# A Comparison of Recruitment Methods for an mHealth Intervention Targeting Mothers: Lessons from the Growing Healthy Program

**DOI:** 10.2196/jmir.5691

**Published:** 2016-09-15

**Authors:** Rachel A Laws, Eloise-Kate V Litterbach, Elizabeth A Denney-Wilson, Catherine G Russell, Sarah Taki, Kok-Leong Ong, Rosalind M Elliott, Sharyn J Lymer, Karen J Campbell

**Affiliations:** ^1^ Deakin University Institute for Physical Activity and Nutrition School of Exercise and Nutrition Science Geelong Australia; ^2^ Centre for Obesity Management and Prevention Research Excellence in Primary Health Care Sydney Australia; ^3^ Deakin University School of Exercise and Nutrition Science Geelong Australia; ^4^ Faculty of Health University of Technology Sydney Sydney Australia; ^5^ La Trobe Analytics Lab La Trobe University Melbourne Australia; ^6^ Faculty of Pharmacy University of Sydney Sydney Australia

**Keywords:** recruitment, mHealth, parents, social media, obesity prevention, infant feeding, children, infants, practitioners, primary health care

## Abstract

**Background:**

Mobile health (mHealth) programs hold great promise for increasing the reach of public health interventions. However, mHealth is a relatively new field of research, presenting unique challenges for researchers. A key challenge is understanding the relative effectiveness and cost of various methods of recruitment to mHealth programs.

**Objective:**

The objectives of this study were to (1) compare the effectiveness of various methods of recruitment to an mHealth intervention targeting healthy infant feeding practices, and (2) explore factors influencing practitioner referral to the intervention.

**Methods:**

The Growing healthy study used a quasi-experimental design with an mHealth intervention group and a concurrent nonrandomized comparison group. Eligibility criteria included: expectant parents (>30 weeks of gestation) or parents with an infant <3 months old, ability to read and understand English, own a mobile phone, ≥18 years old, and living in Australia. Recruitment to the mHealth program consisted of: (1) practitioner-led recruitment through Maternal and Child Health nurses, midwives, and nurses in general practice; (2) face-to-face recruitment by researchers; and (3) online recruitment. Participants’ baseline surveys provided information regarding how participants heard about the study, and their sociodemographic details. Costs per participant recruited were calculated by taking into account direct advertising costs and researcher time/travel costs. Practitioner feedback relating to the recruitment process was obtained through a follow-up survey and qualitative interviews.

**Results:**

A total of 300 participants were recruited to the mHealth intervention. The cost per participant recruited was lowest for online recruitment (AUD $14) and highest for practice nurse recruitment (AUD $586). Just over half of the intervention group (50.3%, 151/300) were recruited online over a 22-week period compared to practitioner recruitment (29.3%, 88/300 over 46 weeks) and face-to-face recruitment by researchers (7.3%, 22/300 over 18 weeks). No significant differences were observed in participant sociodemographic characteristics between recruitment methods, with the exception that practitioner/face-to-face recruitment resulted in a higher proportion of first-time parents (68% versus 48%, *P*=.002). Less than half of the practitioners surveyed reported referring to the program often or most of the time. Key barriers to practitioner referral included lack of time, difficulty remembering to refer, staff changes, lack of parental engagement, and practitioner difficulty in accessing the app.

**Conclusions:**

Online recruitment using parenting-related Facebook pages was the most cost effective and timely method of recruitment to an mHealth intervention targeting parents of young infants. Consideration needs to be given to addressing practitioner barriers to referral, to further explore if this can be a viable method of recruitment.

## Introduction

Mobile health (mHealth) apps hold great promise as an effective delivery mode for evidence-based public health interventions. Interventions using mHealth are appealing for a number of reasons: first, a large proportion of the population has access to (and use) apps on their mobile phones; current data indicate that 75% of the world population has access to a mobile phone [1]. A 2012 global survey reported that mobile phone ownership in the United States encompassed 94% of the population, with similar levels of ownership in the United Kingdom (97%), Australia (86%), China (89%), and India (89%). Smartphone ownership ranges from 60-70% in these countries with the exception of India, where just one in ten people own a smartphone [2]. Second, interest in (and use of) mHealth apps for the management and promotion of health is widespread. Globally, there are over 97,000 health-related apps and approximately 1000 new apps are published every month [3]. A recent survey in the United States reported that 35% of mobile phone users downloaded apps to track or manage their health [4]. Third, evidence suggests that mobile phones are uniquely positioned to bridge gaps in health disparities and enable access to information across demographic groups [5]. Lastly, apps designed for use on mobile phones also provide the advantages of programming flexibility (ie, they can be designed with multiple functions) and can provide *around the clock* high quality information and personalized support, at low cost to both the user and the health provider [6].

As a novel use of emerging technology, mHealth is a relatively new field of research with a need for high quality studies to evaluate the effectiveness of mHealth interventions. Conducting mHealth studies presents unique challenges for researchers, one of which is understanding how best to recruit and retain participants [7], and whether new or novel recruitment methods are required. Recruiting an adequate sample size in a timely and cost effective manner is a critical issue, as it ensures that studies are adequately powered to measure effects and are conducted in an efficient manner [7,8]. Furthermore, the recruitment of diverse samples, including typically under-represented groups, is important to improve the external validity of mHealth studies [7,8].

To date, mHealth studies have used a range of recruitment approaches, including online (advertising on search engines, websites, online forums, direct emails, and social media) and traditional methods of recruitment (flyers, newspaper ads, billboards, TV and radio ads, and direct mail) [7]. Another method of recruitment that may be appropriate for health-related interventions is referral by health practitioners who feel that particular mHealth programs are trusted and fit well with their practices. While Internet-supported therapeutic interventions are gaining popularity [9], there is a lack of studies reporting on the outcomes of practitioner referral to mHealth programs.

There is a paucity of research reporting on recruitment to mHealth programs [7]. We were only able to identify one other study [10] that reported on the reach and cost of various methods of recruitment to an mHealth program. This study did not compare participant characteristics by recruitment method, which is an important issue given the potential differential reach of online versus more traditional methods of recruitment. More is known about recruitment to web-based interventions, with a number of studies [11-20] reporting that the use of online recruitment strategies such as Facebook, search engine advertisements, and promotion on relevant websites, are effective. However, only a few studies of web-based interventions [11,14,15,21] have compared the reach and costs of using online versus traditional methods of recruitment, with conflicting findings. Furthermore, while one study [15] reported no difference in participant characteristics by recruitment method, others [11,14,21] reported online recruitment to be more effective in recruiting *hard to reach* or more *at risk* groups. More research is needed to understand both the reach and costs of various methods of recruitment to both web-based and mHealth interventions, in order to inform the design of future trials.

We have recently developed an mHealth intervention for parents of young infants (growing healthy) that encourages healthy infant feeding practices, with a focus on socioeconomically disadvantaged parents. The program consists of an app and website [22], providing parents with a *one-stop shop* for evidence-based advice and strategies that are consistent with national guidelines pertaining to infant feeding in the first nine months of life. Participating parents received three push notifications or short message service (SMS) text messages (for those without smartphones) regarding infant feeding and related topics, which were relevant to the age of their infant, for each week of the intervention. Messages were tailored to parents’ feeding mode (breast, formula, or mixed feeding) with links to more information on the app or website. Further details about the development of the program have been published elsewhere [23]. A feasibility study of the program has been conducted [23] using a quasi-experimental design, with an mHealth intervention group and a concurrent nonrandomized comparison group. The study used a range of recruitment methods, including traditional approaches (face-to-face recruitment by researchers), practitioner referral, and online recruitment, providing a unique opportunity to examine the reach and costs of these various recruitment approaches.

The aims of this paper were to (1) compare the recruitment rate, costs, and characteristics of participants recruited using a range of recruitment approaches to an mHealth intervention targeting parents with young infants, and (2) to explore factors influencing practitioner referral to the program. These analyses will provide important new insights into recruitment to mHealth interventions, and further our understanding of utilizing health practitioners for referral to such programs.

## Methods

### Study Design and Sample Size

The methods of this study have been published previously [23]. Briefly, the growing healthy study utilized a quasi-experimental design with an mHealth intervention group and a concurrent nonrandomized comparison group. The study aimed to recruit approximately 200 parent/child dyads to the intervention arm and a similar number to the comparison arm, with a focus on recruiting parents from socioeconomically disadvantaged regions. As this was a feasibility study, the sample size was not based on a statistical power calculation; rather, the purpose of the study was to test implementation feasibility, and sample size was therefore tailored to logistical limitations of the time and funds available to support recruitment. The data gathered in this study will provide evidence to guide sample size calculations for a subsequent randomized controlled trial. This paper will focus on recruitment to the intervention arm; details of recruitment to the comparison arm have been published [23].

### Recruitment Methods - Growing healthy Program

**Figure 1:**
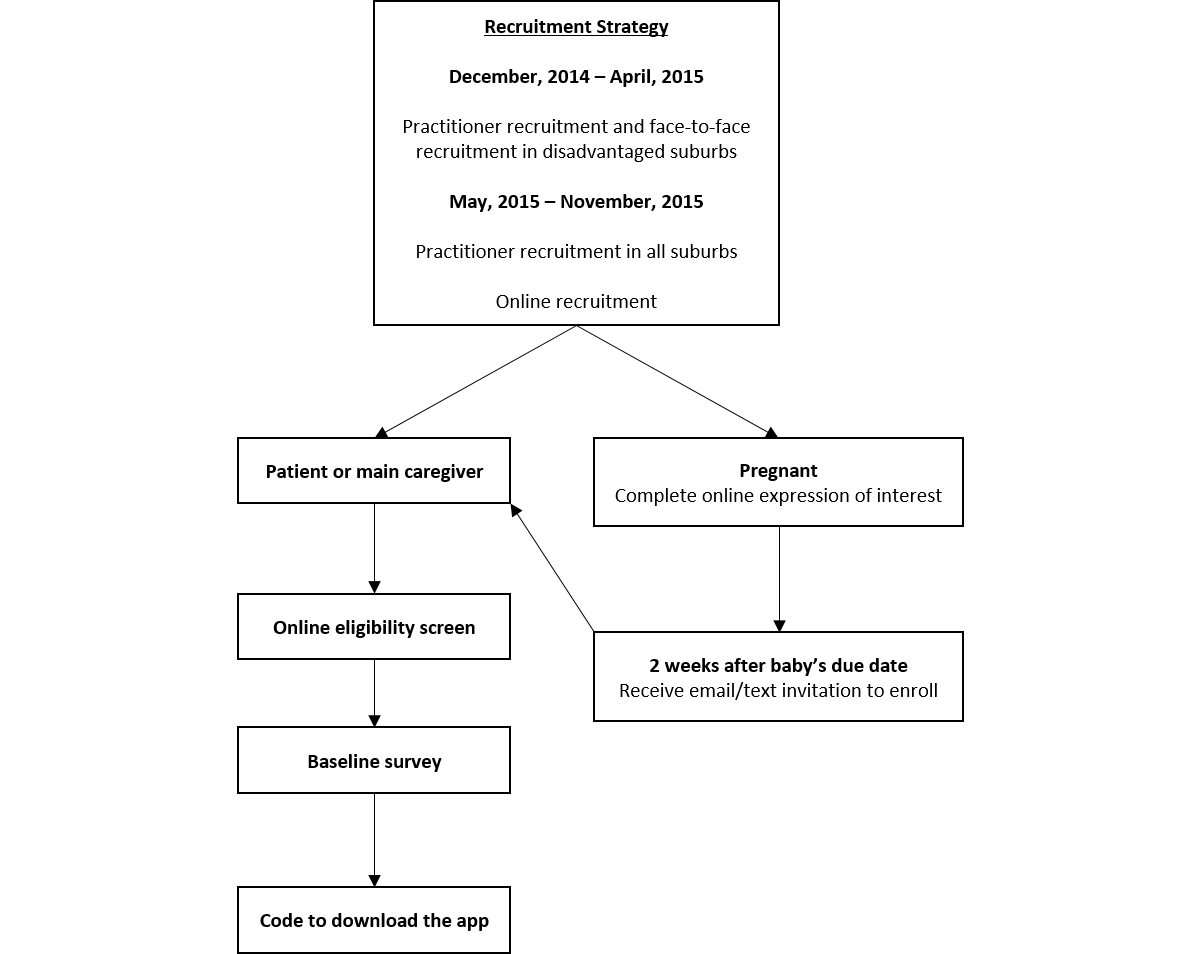
Overview of the recruitment and enrolment process for the Growing healthy program.

Eligibility criteria for participation in the study included: expectant parents (>30 weeks of gestation) or parents/primary care giver with an infant <3 months old, ability to read and understand English, own a mobile phone, ≥18 years old, and living in Australia. Participants were excluded if their infant was born prematurely (before 37 weeks) or had a disability with the potential to impact on infant feeding. Recruitment to the growing healthy program commenced in December, 2014 and continued for a 12-month period. The initial method of recruitment was via practitioner referral and entailed face-to-face recruitment only. However, due to the slow rate of recruitment using these approaches over the initial 6-month recruitment period, online advertising was used in the final six months of recruitment, with the aim of boosting enrolments. Further details of these recruitment methods are described below and outlined in Figure 1.

**Practitioner recruitment methods.**

- Handing out program brochures at routine appointments
- Displaying posters in waiting rooms in participating clinics/centers/practices
- Asking interested parents to complete an expression of interest form (MCH Services only). Using these forms, parents provided contact details and gave permission for the research team to email information about the study directly to them. This approach was used in response to MCH nurses’ concerns that interested parents with young infants may need a reminder to enroll
- Sending a letter of invitation from the practice (general practice only) inviting eligible potential participants (women registered in the practice in final trimester of pregnancy or having an infant less than three months old) to enroll in the program

#### Practitioner Referral

Practitioners were engaged in recruiting parents to the growing healthy program from three primary health care settings: (1) Maternal and Child Health (MCH) nurses (n=87) in two local government areas in Melbourne, Victoria; (2) midwives (n=10) from outpatient antenatal services at a large Melbourne hospital; and (3) practice nurses (n=8) from four general practices in the Illawarra/Shoalhaven Medicare location in New South Wales. Study sites were selected if they had a high relative level of socioeconomic disadvantage in the surrounding communities (based on the Index of Relative Socio-Economic Advantage and Disadvantage [24]) as well as a relatively high birth rate, and if the sites had been involved in previous studies and were within reasonable proximity to the study researchers. Practitioners attended a face-to-face briefing session with the research team, which included a demonstration of the growing healthy app and recruitment strategies. Practitioners were also offered a code to download the app. Practitioners were requested to promote the program to potential participants using one or more of the methods outlined in Textbox 1.

The ethics approval for practice nurses in general practice was conditional on the use of passive recruitment strategies only (ie, display of posters/brochures in waiting areas of practices and sending letters to potential participants). The use of more active approaches, such as practice nurses providing a brochure to a potential participant, were considered by this committee as potentially coercive due to the existing practitioner-patient relationship. However, ethics committees overseeing the study for MCH nurses and midwives approved both passive and active promotion by practitioners, with the condition that practitioners emphasized that the choice to participate was completely voluntary and would not affect the care provided.

Within the MCH Services in Victoria, only practitioners working in the most disadvantaged communities (defined as having an Index of Relative Socio-Economic Advantage and Disadvantage score of less than a 1000 [24]) were initially invited to recruit parents to the program (December, 2014 - April, 2015). However, due to the slow rate of recruitment over the first five months, all MCH nurses working in the area were subsequently invited to recruit parents to the program for the remaining seven months of recruitment (May, 2015 - November, 2015).

#### Face-To-Face Recruitment

Within participating MCH Services in Victoria, a research assistant attended first-time parent groups (n=22, two to eight parents per group) in selected low socioeconomic suburbs to inform parents of the growing healthy program. Recruitment involved providing parents with a copy of the program brochure and collecting the names and email addresses of interested parties. These parents were subsequently emailed a web link inviting them to enroll in the program.

#### Online Recruitment

**Figure 2:**
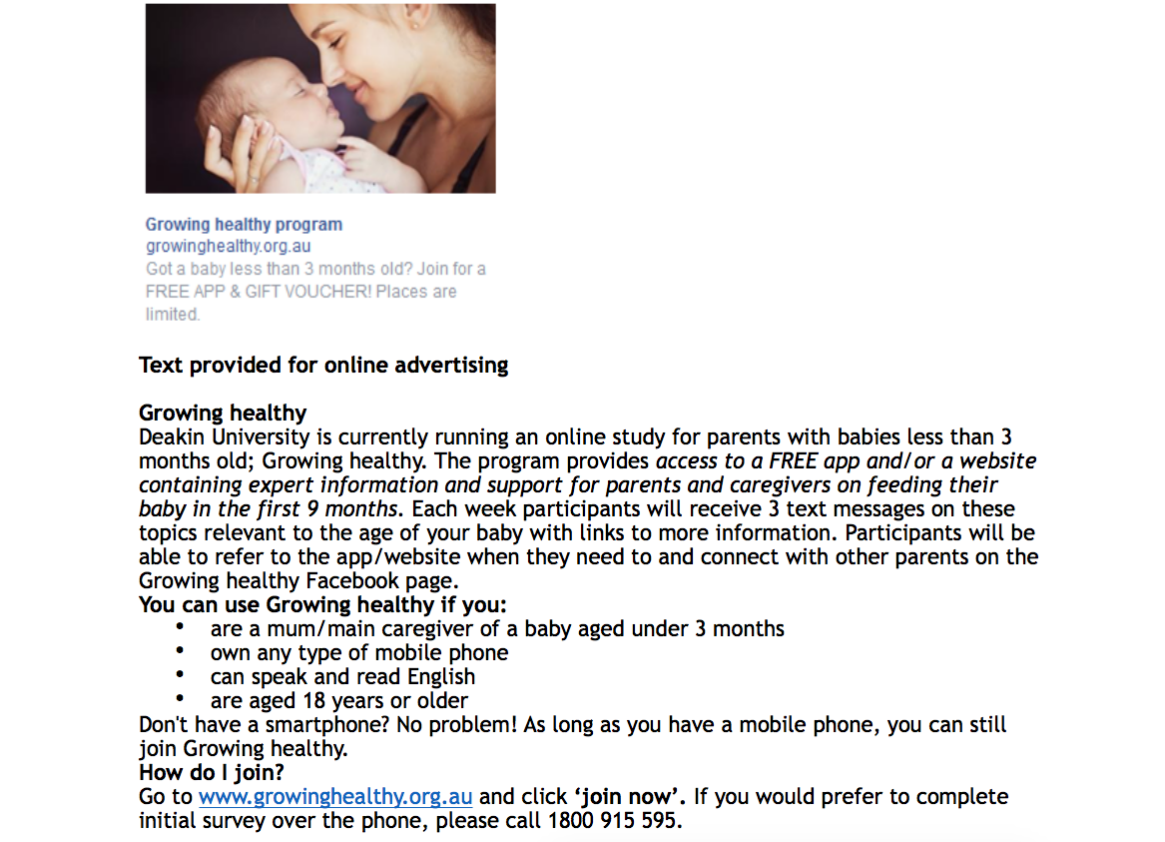
Facebook advertisement.

Online recruitment for the intervention arm commenced in May, 2015 in response to the slow rate of recruitment using practitioner and face-to-face approaches. Online methods involved advertising the program on a range of popular Australian parenting websites and forums, including one advertisement on a parenting website and five Facebook status updates on Facebook pages targeting (and widely followed by) parents of young children. The main factors influencing the choice of website/Facebook pages included: Australian-based groups, groups having a large number of followers, and the advertising costs being within the project budget (costs of advertisements ranged from AUD $65 to $440 each, with costs totaling $832). Additionally, a capped price (AUD $200) official Facebook advertising package was purchased. This initiative ran for five days and could only be seen by those who were using Facebook on a computer (rather than a mobile device) on the side bar of a Facebook newsfeed. Facebook advertising on mobile devices was not possible because the project did not have a public Facebook page established, which is a requirement for Facebook advertising on mobile devices. Figure 2 shows an example of a Facebook advertisement and the text used in online advertising.

In total, online advertisements ran for eight weeks. The snowball effect of social media (ie, people tagging friends and families in the Facebook post) continued to promote the app, resulting in recruitment of a substantial number of parents for a number of weeks following the completion of active advertising. A record of the online recruitment processes was kept, including a list of the date and site of each of the online advertisements and the associated costs.

### Participant Enrolment and Data Collection

Participants enrolled via the program website [22], which involved completing an eligibility screening form, providing consent, and completing a baseline survey that included questions regarding sociodemographic information and how participants heard about the study [23]. To compensate participants for the time involved in completing this survey, participants received an AUD $20 gift voucher. Participants received a code to download the app (at no cost) from the App Store (iPhone users) or Google Play (Android users), or a login for the website (for those without a smartphone capable of supporting the app). Pregnant women that were interested in participating in the study registered their interest on the study website. These mothers immediately received an SMS text message/email inviting them to enroll in the study upon the birth of their baby; a reminder SMS text message/email was sent two weeks after their baby’s due date.

### Recruitment Costs

Several pieces of information were collected to calculate the costs for each recruitment method, as outlined in textbox 2.

Researcher time consisted mainly of research assistants (AUD $42 per hour), but also involved an administrative assistant (AUD $22 per hour) and a PhD student (AUD $14 per hour). Two research fellows (AUD $59 per hour) led the recruitment of practitioners and were involved in the briefing sessions, along with a senior researcher (AUD $70 per hour). All costs were inclusive of *on-costs* such as superannuation and leave, and based on salary scales at Deakin University and University of Technology Sydney. The total costs were calculated for each recruitment method and divided by the number of participants recruited using each method, to calculate a cost per enrolled participant for each type of recruitment. Sensitivity analysis was conducted regarding the costs of researcher time.

**Costs associated with each recruitment method.**

*Practitioner referral:*

• costs of recruitment materials provided to practitioners, researcher time for attending the briefing session, emails to parents expressing interest, and sending of invitation letters from practices. The costs of the practitioners’ time spent recruiting were not included because this was provided *in kind* as part of practitioners’ routine practice

*Face-to-face recruitment:*

• Researcher time for travel and attending first-time parent groups, sending of follow-up emails, and travel costs

*Online recruitment:*

• Researcher time spent searching for, and placing advertisements in, relevant parenting websites and forums, and direct advertising costs

### Practitioner Survey and Interviews

Nine months after recruitment commenced, all participating practitioners were invited to complete a short five-to-ten minute online survey. The survey asked about how frequently the practitioners referred to the program, the methods used in promoting the program, perspectives on the barriers to referring participants, views about the program content and credibility, and perceptions of the sustainability of continued referral of parents to the program. Practitioners who were invited to complete the survey, but had not yet done so, were sent a reminder email on three separate occasions. Practitioners participating in the survey, along with their service managers, were also invited to participate in individual semi-structured telephone interviews to further explore issues pertaining to referral to the program.

### Data Analyses

Differences in participants’ characteristics (obtained from baseline survey) between recruitment methods were tested using Pearson’s Chi-square test statistics for categorical data, and independent t-tests for normally distributed continuous variables using IBM SPSS Statistic version 22 [25]. Recruitment rates were calculated for each recruitment method as the number of eligible participants divided by recruitment time, and an exact 95% confidence interval was calculated based on the relationship between Poisson and chi-square distributions [26].

Practitioner survey data were analyzed descriptively using Microsoft Excel. Practitioner interviews were audio recorded (with permission) and transcribed verbatim. Transcripts were analyzed thematically by one author (RL), using NVivo 10 [27] to organize codes. Codes were cross checked by another author (EL) for consistency in coding approach and interpretation. Minor differences were noted in coding, each of which was resolved through discussion.

### Ethics and Study Approvals

This study was approved by Deakin Human Research Ethics Committee (HREC) (2014_093), University of Technology Sydney HREC (ETH15-0110 for New South Wales participants) and the Victorian Department of Education and Training.

## Results

### Enrolment Status

A total of 585 individuals commenced the baseline survey; 171 (29.2%) failed to complete the survey and 32 participants completed it twice (duplicate surveys). A further 82 (82/585, 14.0%) individuals were considered ineligible (mainly because their baby was older than three months), resulting in 300 enrolled participants (51.3% of those who commenced the baseline survey).

### Rates and Costs of Various Recruitment Strategies

Just over 50% of participants (151/300) were recruited online. This approach proved to be the quickest and cheapest method of recruitment, at an average cost of AUD $14 per participant recruited over a 22-week period (Table 1). Of the online sources of recruitment, advertising on the Facebook pages of popular parenting websites recruited the most participants at the lowest cost (Table 2). An official Facebook advertisement was less successful, resulting in 150 clicks and five participants recruited, despite reaching over 16,000 women of a child bearing age.

Overall, 29.3% of participants (88/300) were recruited by practitioners during a time span of just under one year, with most participants being recruited by MCH nurses (70/88) and few being recruited by practice nurses (16/88) or midwives (2/88). The costs for each participant recruited was AUD $586 for practice nurses, AUD $268 for midwives, and AUD $77 for MCH nurses (Table 1). In terms of face-to-face recruitment, a total of 22 first-time parent groups were visited by the research team over an 18-week period, resulting in 51 expressions of interest and 23 enrolments, at a cost of AUD $100 per participant. Interestingly, 12.7% (38/300) of participants were recruited through recommendations by family and friends, which could have flowed from online or practitioner referrals.

### Comparison of Participant Characteristics by Recruitment Approach

There were no significant differences in participant characteristics by recruitment method, with the exception that participants referred to the program by their practitioner, or recruited by researchers face-to-face, were more likely to be first-time parents/primary care givers compared to those recruited online (Table 3). Approximately 52% (130/251) of participants had no university-level education and approximately one-fifth (53/251) of participants had a high school education or less. There were no sociodemographic differences between participants recruited by practitioners in the first wave of recruitment (disadvantaged suburbs only) compared to those recruited by practitioners in the second wave (all suburbs in the selected areas, data not shown).

**Table 1:** Rates and costs of recruitment strategies.

Recruitment method		Number (%) of participants recruited	Length of recruitment period	Total cost (AUD $)	Cost per participant (AUD $)	Mean number (Confidence Interval) participants recruited per week
**Online recruitment**		151 (50.3)	22 weeks	$2082	$13.79	6.9 (5.8-8.0)
**Practitioner recruitment**						
	MCH^a^ nurse	70 (23.3)	45 weeks	$5371	$76.73	1.6 (1.2-2.0)
	Practice nurse	16 (5.3)	46 weeks	$9371	$585.69	0.3 (0.2-0.6)
	Midwife	2 (0.7)	10 weeks	$536	$268.00	0.2 (0-0.7)
**Face-to-face recruitment**		23 (7.7)	22 weeks	$2310	$100.43	1.0 (0.7-1.6)
**Word of mouth**		38 (12.7)	42 weeks	n/a	n/a	0.9 (0.7-1.4)

^a^ MCH: Maternal and Child Health.

**Table 2:** Online sources of recruitment (n=151) for the growing healthy program.

Source	Number (%)	Total cost (AUD $)	Direct advertising cost per participant (AUD $)
Parenting website Facebook pages	102 (67.5)	$650	$6.37
Facebook advertising	5 (3.3)	$200	$40
Online mothers group Facebook pages	40 (26.5)	$182	$4.55
Facebook (unspecified)	3 (2.0)	n/a	n/a
Internet search	1 (0.7)	n/a	n/a

**Table 3:** Comparison of participant characteristics by recruitment strategy for the growing healthy (mHealth) intervention (n=262).

Participant characteristics		Recruitment method		
		Online (n=151)	Practitioner or face to face (n=111)	*P*value
**Participant age in years, mean (SD), range**		30.05 (4.85), 18-46	31.10 (4.50),18-41	.08
**Country of birth, n (%)**				
	Australia	133 (88.1)	89 (80.2)	.12
	New Zealand	3 (2.0)	3 (2.7)	
	United Kingdom	5 (3.3)	2 (1.8)	
	Other	10 (6.6)	17 (15.3)	
**Aboriginal or Torres Strait Islander, n (%)**				
	Yes	5 (3.3)	1 (0.9)	.20
	No	146 (96.7)	110 (99.1)	
**Marital status, n (%)**				
	Married	111 (73.5)	83 (74.8)	.70
	Defacto relationship	32 (21.2)	25 (22.5)	
	Separated	1 (0.7)	0 (0)	
	Divorced	0 (0)	0 (0)	
	Never married	7 (4.6)	3 (2.7)	
	Widowed	0 (0)	0 (0)	
**Average household gross weekly income (AUD $), n=223, n (%)**				
	Below average (<$1000/week)	16 (11.8)	15 (17.2)	.08
	Average ($1000-<$1500/week)	51 (37.5)	21 (24.1)	
	Above average ($1500-<2000/week)	30 (22.1)	29 (33.3)	
	Higher income (>$2000/week)	39 (28.7)	22 (25.3)	
**Highest level of education n=251, n (%)**				
	High school education or less	30 (21.0)	23 (21.3)	.96
	Trade/certificate/diploma	43 (30.1)	34 (31.5)	
	Degree or higher degree	70 (49.0)	51 (47.2)	
**Employment status, n (%)**				
	Keeping house and/or raising children full time	130 (86.1)	90 (81.1)	0.23
	Working full time	8 (5.3)	12 (10.8)	
	Working part time/casual	8 (5.3)	5 (4.5)	
	Studying (full or part time)	4 (2.6)	1 (0.9)	
	Unemployed/laid off	1 (0.7)	3 (2.7)	
**Health care card, n (%)**				
	Yes	29 (19.2)	13 (11.7)	.10
	No	122 (80.2)	98 (88.3)	
**Self-rated health status, n (%)**				
	Excellent	14 (9.3)	11 (9.9)	.19
	Very good	58 (38.4)	52 (46.8)	
	Good	67 (44.4)	35 (31.5)	
	Fair	12 (7.9)	13 (11.7)	
	Poor	0 (0)	0 (0)	
**Smoking status, n (%)**				
	Never smoked	95 (62.9)	70 (63.1)	.69
	Past smoker	48 (31.8)	32 (28.8)	
	Smoke occasionally	4 (2.6)	3 (2.7)	
	Smoke regularly	4 (2.6)	6 (5.4)	
**Baseline feeding status, n (%)**				
	Breastfeeding	103 (68.2)	65 (58.6)	.19
	Formula feeding	25 (16.6)	20 (18.0)	
	Mixed feeding	23 (15.2)	26 (23.4)	
**First time parent, n (%)**		73 (48.3)	75 (67.6)	.002*

^*^ *P*<.05.

### Feedback from Practitioners - Survey and Interview Findings

A total of 37 of 87 (43%) practitioners completed the online survey to provide feedback on the intervention and the referral process. Four qualitative interviews were subsequently conducted with two MCH nurses (who championed the program in the areas that they worked) and two MCH Service managers in the participating areas. While most practitioners surveyed (76%, 66/87) *agreed* or *strongly agreed* that the growing healthy program was a credible source of information and support for parents on infant feeding, and that there was a need for such programs, less than half of the survey respondents actually referred parents to the program *often* or *most of the time* (Table 4). The primary method of promoting the program was handing out brochures to parents (76%, 66/87) and displaying posters in waiting rooms (60%, 52/87). Only two of the 37 surveyed practitioners showed parents the app on their own device, and only six showed parents the website. Interestingly, only six of the 37 practitioners surveyed had downloaded the app. Nearly half (43%, 37/87) reported not downloading the app or viewing the website (Table 4). A number of practitioners reported problems downloading the app, and (as discussed in the qualitative interviews) this may have affected nurses’ confidence in the program:

We had real problems with the app. So for the nurses to feel confident about recruiting people when the app wasn’t working and getting on to the site, was quite difficult…

Practitioner 4

The survey indicated that a lack of time was the main reason for not referring patients to the app, and this was also discussed in the qualitative interviews:

We often get asked to do so many things and we also have quite a number of tasks that we need to achieve under each of the key age and stage visits anyway.

Practitioner 2

Other barriers included practitioners finding it difficult to remember to refer to the app (43%, 37/87), and the study enrolment process itself, such as the number of steps involved in accessing the app (28%, 24/87; Table 4). Qualitative interviews also highlighted that staff changes, including the use of casual relief staff, was a barrier at one site. During the interviews practitioners also indicated that parents were not always responsive to a referral due to the demands of having a newborn baby, and the lack of uptake or uncertainty about uptake was disheartening, as discussed by one nurse:

Once the baby is here, there’s sleep deprivation, they’re busy and they’re coming to Maternal and Child Health and getting lots of different information. So maybe there’s a bit of information overload…I just remember feeling a bit dejected that people weren’t using it.

Practitioner 3

During the interviews, practitioners made a number of useful suggestions for improving program promotion in the future. These suggestions included engaging parents early (such as targeting parents antenatally when they had more time to engage and download the app), offering the app to parents with babies and toddlers, as well as the use of fridge magnets (instead of paper brochures) to act as ongoing visible reminders for parents to enroll in the program. One interviewee highlighted that vulnerable parents frequently change mobile phones and phone numbers, and that this may be a barrier to parent participation in mHealth programs. Suggestions made to improve practitioner promotion of the program included the integration of promotional materials into the standard *information pack* provided to parents by nurses at each visit, whole of service recruitment (rather than recruitment in selected suburbs), and to provide regular reminders to nurses to promote the program. The use of SMS text reminders or a nurse version of the app that provided nurse-specific push notifications was suggested. Managers also discussed the importance of broader endorsement of the program by the MCH Service funding body, for promotion of the program to become standard practice, as well as the engagement of other local health and social services in promoting the app.

**Table 4:** Practitioner feedback on program promotion (survey findings).

Promotion of Program		Number (%)
**Frequency of promotion or referral (n=37)**		
	Most of the time (more than 75% of eligible parents)	4 (11)
	Often (51-75% eligible parents)	12 (32)
	Sometimes (26-50% eligible parents)	11 (30)
	Rarely (1-25% of eligible parents)	8 (22)
	Never	2 (5)
**Main reason never or rarely promoted the program (n=9)**		
	Sometimes no program materials	1 (11)
	Lack of time	4 (44)
	Do not see parents personally	2 (22)
	Did not like brochure with bottle feeding baby	1 (11)
	No reason	1 (11)
	Not interested	1 (11)
	Most clients have poor English skills	1 (11)
**Views about the referral process (agree or strongly agree)**		
	I had adequate information about the program in order to refer parents (n=30)	27 (90)
	I found it difficult to remember to refer parents to the program (n=30)	13 (43)
	I found the referral process worked well (n=30)	16 (53)
	Some parents found it difficult to enroll in the program (n=29)	8 (28)
**Method of promotion (n=37)**		
	Gave brochures to parents	28 (76)
	Posters in waiting room or clinic areas	22 (60)
	Asked interested parents to complete expression of interest	12 (32)
	Showed parents the website	6 (16)
	Showed parents the app	2 (5)
	Discussed/encouraged in first-time parent groups	3 (8)
	Brochure in waiting room	1 (3)
	Supported staff to show parents the website	1 (3)
	Did not promote the program	2 (5)
**Encountered problems referring parents to program (n=30)**		5 (17)
**Practitioner access to program (n=35)**		
	Downloaded the app	2 (6)
	Viewed the website	13 (37)
	Downloaded app and viewed website	4 (11)
	Neither downloaded app or viewed website	16 (46)

## Discussion

To our knowledge, this is the first study to report on recruitment outcomes for an mHealth intervention targeting parents of young infants, and is unique in comparing outcomes of online versus practitioner-led recruitment. We found online recruitment using parenting-related Facebook pages to be a more effective method of recruitment compared to practitioner-led referral that took more than twice as long, and contributed only 29.3% (88/300) to the total sample, at a substantially higher cost than online recruitment. Face-to-face recruitment by researchers recruited less than 10% (22/300) of the sample, was time consuming, and resulted in higher recruitment costs than online recruitment.

Our findings suggest that consideration should be given to online recruitment strategies in future mHealth interventions (particularly those targeting parents). In particular, strategies using social media such as parenting-related Facebook pages appear particularly successful. There is growing evidence that parents are high users of social media platforms for parenting information and support [28-30], and use online resources as their primary source of lifestyle information [31]. The limited success of the official Facebook advertisement may reflect the fact that the advertisement did not appear on mobile devices, and was only advertised for a one-week period. With recent statistics [32] indicating that more than 50% of people only use Facebook on their mobile phones, this may have been a major limiting factor. This study did not use other forms of social media, such as Twitter or other online advertising (ie, search engine advertisements), so the effectiveness of these approaches in recruiting parents to mHealth interventions remains untested.

A key challenge for mHealth researchers is keeping abreast of the latest online recruitment options. A recent technological development called ResearchKit [33] may help with this problem. ResearchKit is open-sourced and agnostic to the type of mobile operating system being used. As a result, mHealth projects leveraging ResearchKit can reach out to millions of mobile phone users in a short period of time, because ResearchKit natively brokers the recruitment target using the demographic information that individual mobile operating systems already have from their users. This system also reduces costs in a number of ways. First, the cost to develop the technology to support the mHealth recruitment process is reduced. Second, the overall cost of other aspects of recruitment is also reduced, as ResearchKit manages informed consent and facilitates the capture of digital health data. In summary, the open-sourced and platform-agnostic nature of ResearchKit eliminates the need for individual mHealth research projects to *reinvent the wheel* on common technological components of recruitment, and natively allows access to a larger population in which recruitment is carried out.

There were a number of factors that may have reduced the effectiveness of practitioner-led recruitment in this study. There was a two-month delay between the practitioner briefing session and commencement of recruitment due to a technical issue with the data collection system. It is quite likely that recruitment would have been more successful if briefing coincided with the start of recruitment. There were also delays in program brochures being distributed to the nurse teams by the service managers, due to more pressing staff issues. There was only limited email contact between the research team and practitioners during the recruitment period due to staff time pressures, providing few opportunities to remind practitioners about the study. These circumstances reflect the reality of working with busy primary health care practitioners who were asked to add program recruitment to their long list of issues to cover in appointments with parents. Furthermore, parents recruited by practitioners were required to remember to go to the study website to enroll, whereas online participants were able to do this at the *click of a button* directly from the online advertisement. The findings from our survey with practitioners also highlighted that very few practitioners actually showed parents the website or app to help promote the program, in contrast to some of our online advertising in which a picture of the app homepage was visible. Over 40% of practitioners surveyed had not downloaded the app or viewed the website, which may reflect a lack of time, interest, technical problems experienced, and possibly the age of the nurses (with nearly 70% over 50 years of age [34]). These issues reflect the findings of recent research that reported a lack of familiarity with mHealth technologies, fear of loss-of-information control, concern about practitioner-patient relationships, and medicolegal risks to be amongst the barriers limiting antenatal health professionals from engaging with mHealth programs [35,36].

Recruitment rates by practice nurses in this study were very low, and as a result costs per participant recruited were high, partially reflecting the large amount of researcher time involved in recruiting and engaging practices. At the time of the study, primary health care organizations were undergoing a restructuring, making it difficult to engage general practices. As a result, only four practices and eight practice nurses were recruited. Furthermore, ethical requirements prevented nurses from directly promoting the program to parents; instead promotion of the program occurred via direct mail to potentially eligible parents, and displays of the program brochure and poster in clinic waiting areas. In contrast, over 80 MCH nurses were involved in the study, and were able to directly promote the program, resulting in higher yields and reduced costs per participant. The organization of MCH Services also allowed for more time-efficient briefing and engagement with practitioners. There was potentially higher buy-in amongst MCH nurses, given the close alignment of the program with their service goals compared to practice nurses who have a generalist role.

Despite our finding that online recruitment was less costly than practitioner-led recruitment, primary health care practitioners are still likely to have an important role to play in promoting and reinforcing mHealth interventions to parents, because parents access these services so frequently. Data from Victoria, Australia suggest that on average parents make 11 visits to general practitioners and 14 visits to MCH nurses in the first year of their child’s life, and most of these visits are unrelated to illness [37]. The findings from this study suggest that promotion of mHealth interventions need to be integrated into standard procedures to become a streamlined part of routine practice. Practitioners also need regular prompts and reminders to engage with parents using such interventions. Consideration should be given to mobile forms of reminders, such as SMS texts or a practitioner version of the app with tailored reminders. Broader endorsement of mHealth programs by practitioner governing and funding bodies is also required for promotion of such programs to become standard practice. The cost of practitioner-led recruitment could also be decreased by broader promotion of the program to practitioner governing bodies and associations, and online promotional videos rather than face-to-face briefing sessions.

Interestingly, despite practitioner recruitment initially occurring in more disadvantaged areas, there were no significant differences in the measured sociodemographic characteristics between participants recruited online versus those recruited by their practitioner or face-to-face by researchers. There was, however, a higher proportion of first-time parents recruited using practitioner-led and face-to-face recruitment approaches. This finding likely reflects face-to-face recruitment at first-time parent groups and practitioners having greater contact with first-time parents and/or more promotion of the program to these parents. Interestingly, more than half of those who enrolled online were second time parents, suggesting a high demand for the program beyond first-time parents. A number of early obesity prevention interventions [38-41] have targeted only first-time parents, with the rationale that the intervention effect may be larger in parents with no previous experience with infant feeding, although this remains untested and will be explored in the outcomes of this study.

The findings of this study are largely in line with the TXT2BFiT mHealth trial [10] that targeted weight gain prevention in adults 18-35 years of age. This study also reported a slow rate of recruitment via health practitioners (in this case general practitioners). The TXT2BFiT trial also found that paid official Facebook advertisements resulted in low uptake and high cost compared to other online advertising, which concurs with our findings. Our findings are also in line with some previous research of web-based interventions (mainly in the smoking cessation field) that have demonstrated that online advertising produces a greater yield of participants [11,14,15,21] than traditional methods of recruitment, and at a lower cost [11,15,21]. There are conflicting findings regarding the impact of recruitment strategies on participants’ baseline characteristics. In findings similar to ours, a previous study examining smoking cessation [15] also found no significant differences for baseline characteristics between recruitment type (traditional versus online recruitment). However, other studies have reported online recruitment to be more effective in recruiting *hard to reach* or more *at risk* groups than traditional recruitment approaches [11,14,21].

This study has a number of strengths and weaknesses. The findings of the study are relevant to the recruitment of parents of young infants to mHealth interventions, and cannot be generalized outside of this context. Different research staff were involved in various recruitment strategies (eg, online recruitment only involved a research assistant, while practitioner recruitment involved more senior researchers to promote practitioner buy-in). While this factor may influence the interpretation of costings, a sensitivity analysis revealed no difference in costing outcomes when only research assistants were used in the costing calculations. The study relied on self-reports of how participants heard about the study, and this may be subject to recall bias. However, because recruitment via practitioners occurred in specific geographical areas and commenced prior to any online advertising, we were able to cross-reference this data against participant addresses and time of recruitment for clarification in some cases. We were unable to determine if word of mouth recruitment resulted from practitioner or online promotion, and thus participants who said they heard about the study from family or friends were excluded from the costing and comparison of participant characteristics. We did observe a rise in word of mouth recruitment that corresponded with online advertising, suggesting that online recruitment may provide an easy method of referring others (ie, tagging friends/family in Facebook posts).

Another limitation of the study was the low response rate to the practitioner survey, and that only four practitioners agreed to be interviewed. This limitation may have resulted in response bias, with those harboring the strongest views about the app (either positive or negative) being more likely to participate. However, the integration of the survey and qualitative findings is a strength, and together provide additional insights into factors influencing practitioner recruitment. The four practitioners that were interviewed can be considered *key informants*; two of whom were MCH Service managers who were aware of the broader views of MCH nurses in their respective services. The other two participants were practitioner *champions* for the project in their respective services, and again were aware of some of the broader practitioner views about the program via informal discussions with their colleagues. We have yet to test whether there is any difference in retention or outcomes between the various recruitment strategies, and this issue will be a focus for future analyses.

### Conclusion

This study provides new insights into the relative effectiveness of various recruitment strategies for a parenting-related mHealth intervention. Our findings suggest that mHealth interventions targeting parents should consider online methods of recruitment through Facebook pages linked to popular parenting websites, and that this tactic is likely to result in researchers meeting their required sample sizes in a shorter timeframe and at a lower cost. Participant characteristics were similar for practitioner-led and online recruitment, with the exception that participants recruited by practitioners were more likely to be first-time parents. While practitioner-led recruitment took longer and recruited fewer participants at a higher cost, this approach should not be dismissed because of the high reach of primary health care practitioners (particularly those working closely with families) and the potential to reinforce program content. Addressing practitioner barriers to referral, including improving access to the mHealth intervention, regular reminders, and integration into routine practice, is likely to be important in enhancing practitioner recruitment to mHealth programs.

## Abbreviations

HREC: Human Research Ethics Committee
MCH: Maternal and Child Health
mHealth: mobile health
SMS: short message service
